# New Aggregation-Induced Delayed Fluorescence Luminogens With Through-Space Charge Transfer for Efficient Non-doped OLEDs

**DOI:** 10.3389/fchem.2019.00199

**Published:** 2019-04-05

**Authors:** Panpan Zhang, Jiajie Zeng, Jingjing Guo, Shijie Zhen, Biao Xiao, Zhiming Wang, Zujin Zhao, Ben Zhong Tang

**Affiliations:** ^1^State Key Laboratory of Luminescent Materials and Devices, Center for Aggregation-Induced Emission, South China University of Technology, Guangzhou, China; ^2^Key Laboratory of Optoelectronic Chemical Materials and Devices, School of Chemical and Environmental Engineering, Jianghan University, Ministry of Education, Wuhan, China; ^3^Department of Chemistry, Hong Kong Branch of Chinese National Engineering Research Center for Tissue Restoration and Reconstruction, The Hong Kong University of Science and Technology, Hong Kong, China

**Keywords:** aggregation-induced delayed fluorescence, thermally activated delayed fluorescence, through-space charge transfer, organic light-emitting diodes, hexaphenylbenzene

## Abstract

In this work, two tailor-made luminogens comprising of electron donors (acridine and phenoxazine) and acceptor (triazine) bridged by the through-space conjugated hexaphenylbenzene (HPB) are synthesized and characterized. Their thermal stability, electrochemical behaviors, crystal, and electronic structures, and photophysical properties are systematically investigated. The crystal and electronic structures reveal that the peripheral phenyls in HPB are closely aligned in a propeller-like fashion, rendering efficient through-space charge transfer between donor and electron moieties. These molecules display weak fluorescence with negligible delayed component in solutions but strong fluorescence with greatly increased delayed component upon aggregate formation, namely aggregation-induced delayed fluorescence (AIDF). Their neat films exhibit high photoluminescence quantum yields (PLQY), and prominent delayed fluorescence. The non-doped organic light-emitting diodes (OLEDs) based on these new luminogens exhibit excellent performance with maximum external quantum efficiency of 12.7% and very small efficiency roll-off of 2.7% at 1,000 cd m^−2^. Designing AIDF molecules with through-space charge transfer could be a promising strategy to explore robust luminescent materials for efficient non-doped OLEDs.

## Introduction

Organic light-emitting diodes (OLEDs) have been extensively studied owing to their excellent properties, such as flexibility, light weight, energy conservation, and so forth, and have gradually become a highly promising technology for flat panel display and white lighting. Organic electroluminescent materials are the foundation of OLEDs. The first-generation luminescent materials for OLEDs are fluorescent materials, such as tris(8-hydroxyquinolinato)aluminum (Alq_3_) (Tang and VanSlyke, 1987). But the efficiency of the device is greatly limited because only 25% singlet excitons are harvested for light emission in devices. The second-generation luminescent materials are phosphorescent materials that can achieve almost 100% exciton utilization via strong spin orbit coupling of heavy metals such as platinum and iridium (Baldo et al., 1998; Adachi et al., 2001; Sasabe and Kido, 2013; Minaev et al., 2014). However, the scarcity and high price of noble metals greatly increase the device cost. So, the search for efficient and cheap luminescent materials still remains an urgent task. In response to these issues, several alternative strategies have been proposed, among which purely organic materials with thermally activated delayed fluorescence (TADF) have received the most interest (Endo et al., 2009; Gong et al., 2011; Wang et al., 2014; Zhang et al., 2014; Hirata et al., 2015; Seino et al., 2016). The OLEDs based on TADF emitters can extract light from both triplet and singlet excitons via spin-converting reverse intersystem crossing (RISC) under thermal activation, theoretically granting excellent internal quantum efficiencies (IQE) of up to 100% of the devices (Sun et al., 2014; Kaji et al., 2015; Lee et al., 2015a).

Generally, TADF emitters adopt highly twisted electron donor-acceptor (D-A) structures to realize small Δ*E*_ST_, but the PLQY are often reduced, particularly in the aggregated state, which require doping technique in device fabrication. Recently, an interesting strategy to balance Δ*E*_ST_ and PLQY by through-space charge transfer effect has been proposed (Kawasumi et al., 2015; Rajamalli et al., 2016; Chen et al., 2017; Tsujimoto et al., 2017). For example, Wang et al. (Shao et al., 2017) took advantage of through-space charge transfer between D and A to realize blue TADF polymers with a non-conjugated polyethylene backbone. Spatial electronic coupling between D and A units results in small Δ*E*_ST_ (0.019 eV) and high PLQY (60%) in film. The resulting blue polymers exhibited good EL performance with a high EL efficiency of 12.1%. In these materials, D and A are physically separated but are spatially proximate. On one hand, the sufficient separation of highest occupied molecular orbital (HOMO) and lowest unoccupied molecular orbital (LUMO) is achieved, resulting in small Δ*E*_ST_ and thus delayed fluorescence. On the other hand, the electron clouds of D and A can interact with each other via a through-space manner to expedite the radiative transition rate, and enhanced PLQY can be expected.

It is well-known that most TADF emitters need complicated doping technique to suppress emission quenching and exciton annihilation (Tao et al., 2014; Furue et al., 2016). However, severe efficiency roll-off still happens as luminance increases, which impedes their large-scale commercial application (Cao et al., 2017). According to the recent studies, aggregation-induced delayed fluorescence (AIDF) materials provide an advisable strategy for solving this problem (Guo et al., 2017a, 2018a; Huang et al., 2017; Liu et al., 2018). Aggregation-induced delayed fluorescence (AIDF) luminogens are free of concentration quenching, and can exhibit strong delayed fluorescence upon aggregate formation. And the triplet excitons are rapidly converted to singlet excitons by RISC, inducing increased EL efficiency. Furthermore, AIDF luminogens present a highly twisted conformation, which can weaken intermolecular interactions and thus reduces short-range Dexter energy transfer. In consequence, non-doped OLEDs based on AIDF luminogens provide very small efficiency roll-off (Gan et al., 2016; Guo et al., 2017b).

Hexaphenylbenzene (HPB) derivatives have attracted intense research interest because of their fantastic geometry and wide application prospects (Waldvogel et al., 1999; Geng et al., 2001; Balzani et al., 2003; Tanaka et al., 2010; Lambert et al., 2012). HPB not only exhibits strong toroidal delocalization of π-electrons (Sun et al., 2005; Vij et al., 2016) but also has noteworthy aggregation-induced emission (AIE) property (Cho et al., 2006; Kanibolotsky et al., 2010). Integrating various D-A systems into HPB allows us to regulate molecular orbitals distribution, therefore adjust the Δ*E*_ST_ value and realize delayed fluorescence. Based on these considerations, herein, we develop two novel luminogens containing an electron D-A system built on HPB. The D and A moieties are positioned in close proximity so that electron clouds of D and A can communicate with each other through spatial interactions. The designed HPB-based molecules exhibit distinct AIDF property, and furnish efficient non-doped OLEDs with very small efficiency roll-off.

## Experimental

### Synthesis

2-(4-Ethynylphenyl)-4,6-diphenyl-1,3,5-triazine (**2**): Into a 250 mL two-necked round bottom flask was placed compound **1** (7.765 g, 20 mmol), trimethylsilylacetylene (5.653 ml, 40 mmol), Pd(PPh_3_)_2_Cl_2_ (1.404 g, 2 mmol), copper iodide (0.761 g, 4 mmol), and PPh_3_ (1.049 g, 4 mmol). The flask was evacuated under vacuum and flushed with dry nitrogen by three times and a mixed solvent system of tetrahydrofuran (THF) and triethylamine (100 mL, v/v = 1: 3) was injected. The reaction mixture was refluxed for 12 h. After cooling to room temperature, the mixture was poured into water and extracted with dichloromethane by three times. The combined organic layers were dried over anhydrous magnesium sulfate. After filtration and solvent evaporation, a mixture of the resulting crude product, KOH (2.0 g, 35.7 mmol) and K_2_CO_3_ (2.0 g, 14.5 mmol) were added into a mixed solvent system of methanol and THF (60 mL, v/v = 1: 1), and then stirred at room temperature for 12 h. The mixture was poured into water and extracted with dichloromethane by three times. The combined organic layers were dried over anhydrous magnesium sulfate. After filtration and solvent evaporation, the crude product was purified by silica-gel column chromatography (dichloromethane: petroleum ether, v/v = 1: 7). White solid of compound **2** was obtained in 65% yield. ^1^H NMR (400 MHz, CDCl_3_), δ (TMS, ppm): 8.8–8.73 (m, 6H), 7.70 (d, *J* = 8.4 Hz, 2H), 7.66–7.56 (m, 6H), 3.27 (s, 1H); ^13^C NMR (125 MHz, CDCl_3_), δ (TMS, ppm): 171.90, 171.06, 136.63, 136.23, 132.78, 132.53, 129.14, 128.94, 128.83, 126.30, 83.55, 79.88. HRMS (C_23_H_15_N_3_): *m/z* 334.1354 [M + H^+^, calcd 334.1344].

10-(4-((4-(4,6-Diphenyl-1,3,5-triazin-2-yl)phenyl)ethynyl)phenyl)-10-phenoxazine (**5a**): Into a 250 mL two-necked round bottom flask was placed compound **2** (3.331 g, 10 mmol), compound **3** (1.685 g, 5 mmol), Pd(PPh_3_)_2_Cl_2_ (0.351 g, 0.5 mmol), copper iodide (0.190 g, 1 mmol), and PPh_3_ (0.262 g, 1 mmol). The flask was evacuated under vacuum and flushed with dry nitrogen by three times and a mixed solvent system of THF and triethylamine (100 mL, v/v = 1: 3) was injected. The reaction mixture was refluxed for 12 h. After cooling to room temperature, the mixture was poured into water and extracted with dichloromethane by three times. The combined organic layers were dried over anhydrous magnesium sulfate. After filtration and solvent evaporation, the crude product was purified by silica-gel column chromatography (dichloromethane: petroleum ether, v/v = 1: 10). Yellow solid of compound **5a** was obtained in 23% yield. ^1^H NMR (500 MHz, CDCl_3_), δ (TMS, ppm): 8.82–8.78 (m, 6H), 7.81 (d, *J* = 7.0 Hz, 2H), 7.76 (d, *J* = 8.4 Hz, 2H), 7.66–7.58 (m, 6H), 7.38 (d, *J* = 7.4 Hz, 2H), 6.73–6.59 (m, 6H), 5.97 (s, 2H); ^13^C NMR (100 MHz, CDCl_3_), δ (TMS, ppm): 171.90, 171.10, 146.31, 144.09, 136.35, 136.26, 134.56, 134.17, 132.79, 132.06, 131.21, 129.15, 129.08, 128.84, 127.15, 123.43, 121.73, 116.01, 115.72, 113.40, 91.23, 90.70. HRMS (C_41_H_26_N_4_O): *m/z* 590.2086 [M^+^, calcd 590.2107].

10-(4-((4-(4,6-Diphenyl-1,3,5-triazin-2-yl)phenyl)ethynyl)phenyl)-9-9dimethyl-9,10-dihydroacridine (**5b**): The procedure was analogous to that described for **5a**. White solid of compound **5b** was obtained in 25% yield. ^1^H NMR (500 MHz, CDCl_3_), δ (TMS, ppm): 8.83–8.78 (m, 6H), 7.85 (d, *J* = 8.4 Hz, 2H), 7.78 (d, *J* = 8.6 Hz, 2H), 7.66–7.58 (m, 6H), 7.49–7.46 (m, 2H), 7.38 (d, *J* = 8.4 Hz, 2H), 7.02–6.98 (m, 2H), 6.97–6.93 (m, 2H), 6.31 (d, *J* = 8.0 Hz, 2H), 1.7 (s, 6H); ^13^C NMR (125 MHz, CDCl_3_), δ (TMS, ppm): 171.90, 171.12, 141.70, 140.80, 136.29, 136.28, 134.41, 132.78, 132.06, 131.71, 130.30, 129.15, 129.09, 128.84, 127.27, 126.56, 125.45, 123.11, 120.94, 114.17, 91.46, 90.54, 36.15, 31.37. HRMS (C_44_H_32_N_4_): *m/z* 616.2616 [M^+^, calcd 616.2627].

10-(4″-(4,6-Diphenyl-1,3,5-triazin-2-yl)-3′,4′,5′,6′-tetraphenyl-[1,1′:2′,1″-terphenyl]-4-yl)-10H-pheno-xazine (TRZ-HPB-PXZ): Into a 50 mL two-necked round bottom flask was placed a mixture of tetraphenylcyclopentadienone (0.922 g, 2.4 mmol) and compound **5a** (1.180 g, 2 mmol) and then diphenyl ether (15 mL) was added. The mixture was refluxed for 10 h and then cooled to room temperature. Ethanol was added into the mixture to precipitate the product, which was collected and washed with ethanol. Yellow solid of TRZ-HPB-PXZ was obtained in 56% yield. ^1^H NMR (400 MHz, CDCl_3_), δ (TMS, ppm): 8.74–8.69 (m, 4H), 8.47–8.42 (m, 2H), 7.62–7.57 (m, 2H), 7.56–7.51 (m, 4H), 7.15–7.12 (m, 2H), 7.11–7.07 (m, 2H), 6.96–6.87 (m, 20H), 6.84–6.80 (m, 2H), 6.52–6.48 (m, 2H), 6.43–6.30 (m, 4H), 5.48 (d, *J* = 7.8 Hz, 2H); ^13^C NMR (125 MHz, CDCl_3_), δ (TMS, ppm): 171.63, 171.27, 145.67, 144.85, 143.81, 143.79, 141.49, 141.47, 141.13, 140.95, 140.52, 140.40, 140.37, 140.36, 140.32, 140.18, 139.82, 139.72, 136.31, 134.19, 133.33, 132.59, 132.12, 131.63, 131.53, 131.52, 131.49, 129.04, 128.72, 127.53, 127.09, 126.91, 126.90, 126.87, 125.87, 125.66, 125.61, 125.57, 123.31, 115.81, 115.16, 113.20. HRMS (C_69_H_46_N_4_O): *m/z* 946.3687 [M^+^, calcd 946.3672].

10-(4″-(4,6-Diphenyl-1,3,5-triazin-2-yl)-3′,4′,5′,6′-tetraphenyl-[1,1′:2′,1″-terphenyl]-4-yl)-9,9-dimeth-yl-9,10-dihydroacridine (TRZ-HPB-DMAC): The procedure was analogous to that described as TRZ-HPB-PXZ. White solid of TRZ-HPB-DMAC was obtained in 60% yield. ^1^H NMR (500 MHz, CDCl_3_), δ (TMS, ppm): 8.74–8.71 (m, 4H), 8.48 (d, *J* = 8.2 Hz, 2H), 7.61–7.57 (m, 2H), 7.56–7.52 (m, 4H), 7.27 (s, 1H), 7.17 (d, *J* = 8.1 Hz, 2H), 7.12 (d, *J* = 8.0 Hz, 2H), 6.98–6.87 (m, 21H), 6.84–6.79 (m, 4H), 6.61–6.57 (m, 2H), 5.88–5.85 (m, 2H), 1.52 (s, 6H); ^13^C NMR (125 MHz, CDCl_3_), δ (TMS, ppm): 171.52, 171.19, 145.66, 141.05, 140.96, 140.80, 140.55, 140.48, 140.36, 140.32, 140.18, 140.14, 139.97, 139.66, 138.18, 136.24, 133.87, 133.22, 132.46, 132.09, 131.59, 131.47, 131.44, 131.42, 129.77, 129.61, 128.95, 128.62, 127.46, 126.98, 126.81, 126.79, 126.76, 126.30, 125.75, 125.58, 125.50, 125.44, 124.95, 120.19, 113.88, 35.84, 31.23. HRMS (C_72_H_52_N_4_): *m/z* 972.4164 [M^+^, calcd 972.4192].

### X-Ray Crystallography

Crystal data for TRZ-HPB-DMAC (CCDC 1885466): C_72_H_52_N_4_, *M*_W_ = 973.17, monoclinic, C 2/c, *a* = 31.3912(15), *b* = 10.8163(6), *c* = 39.535(3) Å, β = 110.8700(10)°, *V* = 12542.9(13) Å^3^, *Z* = 8, *D*c = 1.031 g cm^−3^, μ = 0.060 mm^−1^ (MoKα, λ = 0.71073), *F*(000) = 4096, *T* = 173(2) K, 2θ_max_ = 25.242° (98.2%), 41548 measured reflections, 11317 independent reflections (*R*_int_ = 0.0914), GOF on *F*^2^ = 1.076, *R*_1_ = 0.1503, w*R*_2_ = 0.1529 (all data), *R*_1_ = 0.0684, w*R*_2_ = 0.1296 [I>2sigma(I)], Δe 0.206 and −0.251 eÅ^−3^.

### OLED Fabrication and Characterization

Glass substrates pre-coated with a 95 nm thin layer of indium tin oxide (ITO) with a sheet resistance of 20 Ω per square were thoroughly cleaned for 10 min in ultrasonic bath of acetone, isopropyl alcohol, detergent, deionized water, and isopropyl alcohol and then treated with O_2_ plasma for 5 min in sequence. Organic layers were deposited onto the ITO-coated substrates by high-vacuum (< 5 × 10^−4^ Pa) thermal evaporation. Deposition rates were controlled by independent quartz crystal oscillators, which were 1 ~ 2 Å s^−1^ for organic materials, 0.1 Å s^−1^ for LiF, and 6 Å s^−1^ for Al, respectively. The emission area of the devices was 3 × 3 mm^−2^ as shaped by the overlapping area of the anode and cathode. All the device characterization steps were carried out at room temperature under ambient laboratory conditions without encapsulation except spectrum collection process. EL spectra were taken by an optical analyzer, Photo Research PR705. Current density and luminance vs. driving voltage characteristics were measured by Keithley 2,420 and Konica Minolta chromameter CS-200, respectively. External quantum efficiencies were calculated by assuming that the devices were Lambertian light sources.

## Results and Discussion

**Scheme 1:**
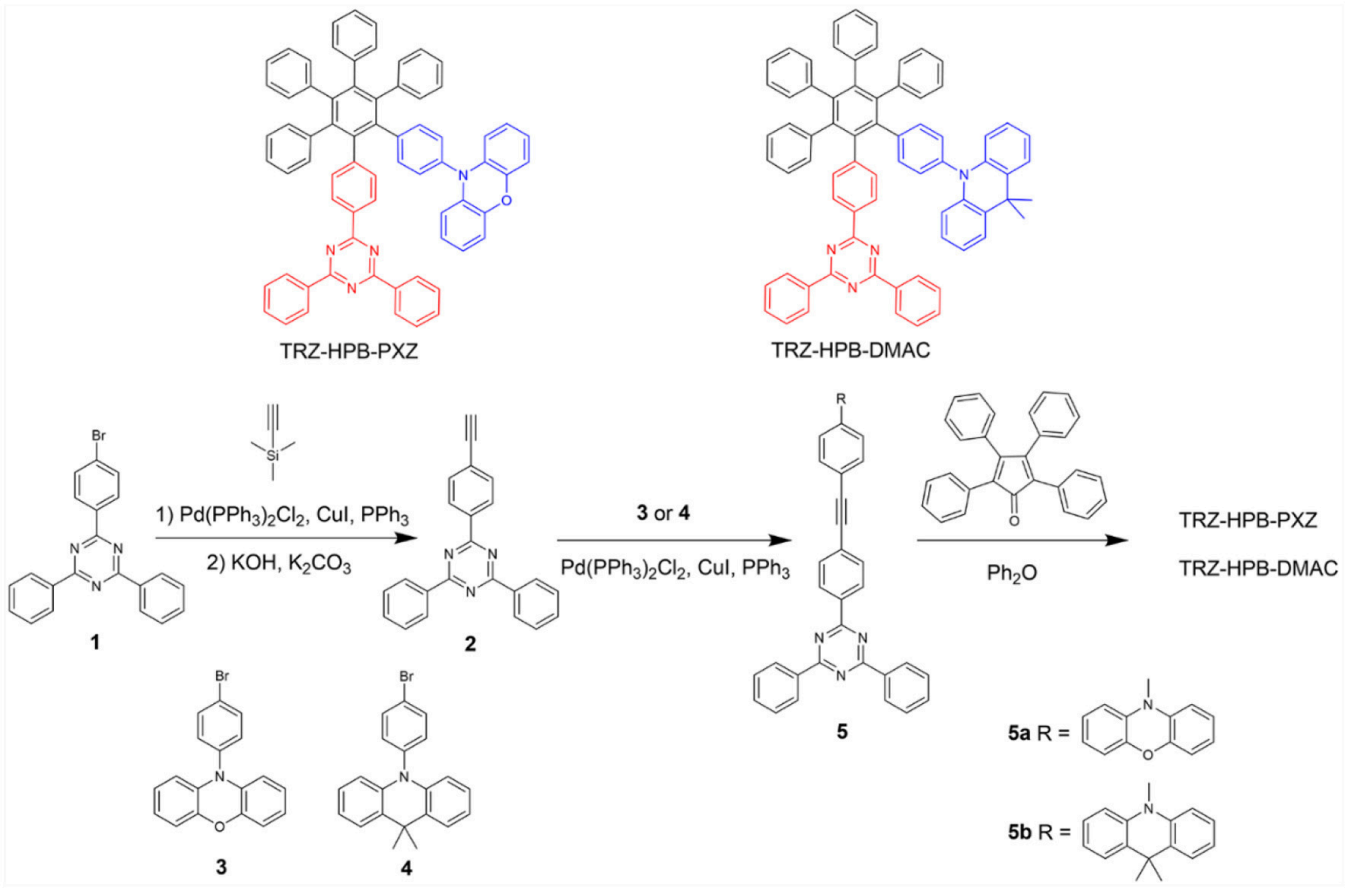
Synthetic routes of TRZ-HPB-PXZ and TRZ-HPB-DMAC.

**Figure 1:**
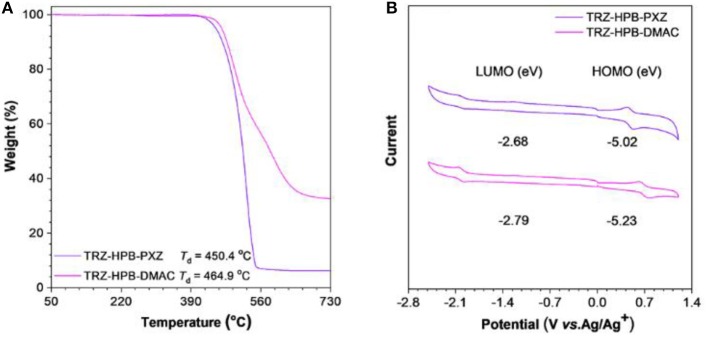
**(A)** TGA curves of TRZ-HPB-PXZ and TRZ-HPB-DMAC, recorded under nitrogen at a heating rate of 20°C min^−1^. **(B)** Cyclic voltammograms of TRZ-HPB-PXZ and TRZ-HPB-DMAC, measured in acetonitrile containing 0.1 M tetra-*n*-butylammonium hexafluorophosphate. Scan rate: 100 mV s^−1^.

The synthetic procedures of these new HPB-based molecules are described in Scheme 1. Intermediates **5a** and **5b** were prepared from compound 1 in two steps by Sonogashira reactions. Subsequently, the target TRZ-HPB-PXZ and TRZ-HPB-DMAC were synthesized through Diels-Alder reactions between **5a** and **5b** with tetraphenylcyclopentadienone, respectively. These HPB-based molecules were identified using ^1^H NMR, ^13^C NMR and high-resolution mass spectroscopy. The thermal stabilities of TRZ-HPB-PXZ and TRZ-HPB-DMAC were evaluated by differential scanning calorimetry (DSC) and thermogravimetric analysis (TGA) methods. They all exhibit good thermal properties with high decomposition temperatures (*T*_d_) of 450.4 and 464.9°C, respectively (Figure 1A). But no glass transition temperatures are observed. The results demonstrate that they are thermally stable and can be used as active layers in OLEDs by vacuum-deposition technique. The cyclic voltammetry was used to test their electrochemical behaviors. They have similar reversible oxidation and reduction processes (Figure 1B), indicative of good electrochemical stability. The HOMO and LUMO energy levels are determined to be −5.02 and −2.68 eV for TRZ-HPB-PXZ, and −5.23 and −2.79 eV for TRZ-HPB-DMAC, respectively.

**Figure 2:**
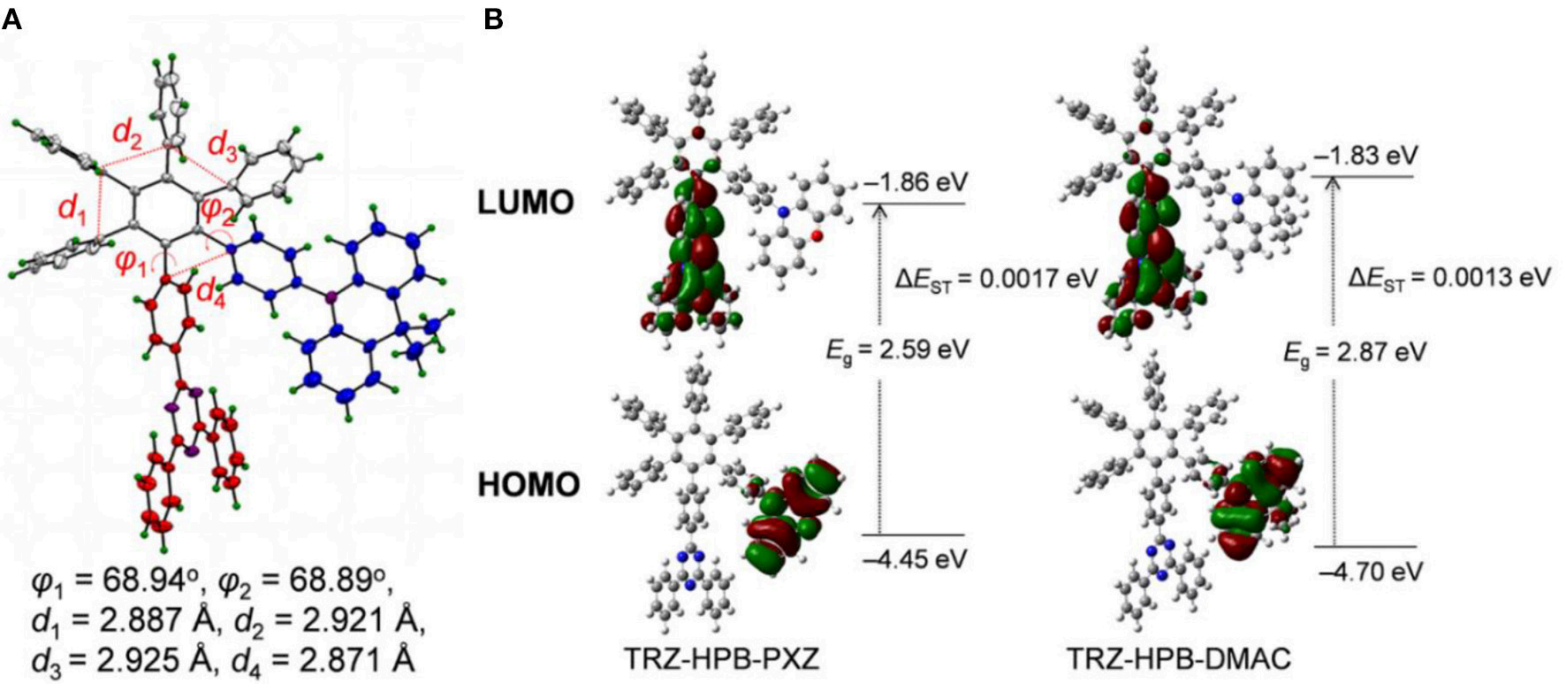
**(A)** Crystal structure of TRZ-HPB-DMAC. **(B)** Spatial distributions of HOMO and LUMO, and Δ*E*_ST_ values of the HPB-based molecules, calculated by DFT/TDDFT method.

The single crystals of TRZ-HPB-DMAC were obtained from the THF-hexane mixture by slow solvent evaporation and subject to crystallography analysis. The crystal structure shows that the dihedral angles between 2,4,6-triphenyl-1,3,5-triazine (TRZ) and the central phenyl is 68.94°, and the dihedral angles between 9,9-dimethyl-10-phenyl-acridine (DMAC) and the central phenyl is 68.89°, implying that through-bond conjugation is relatively weak. The six peripheral phenyl groups of HPB are connected to the central phenyl ring in a propeller-like fashion. They are closely aligned with the shortest distances < 3.0 Å between adjacent phenyls (Figure 2A), indicating that there are strong electronic coupling interactions between these phenyl groups, namely through-space conjugation (Lambert, 2005; Zhen et al., 2018). Furthermore, the shortest distance between TRZ and DMAC is only 2.871 Å, which is close enough to produce intramolecular through-space electronic coupling, providing a charge transfer channel. The screwy architecture is of great importance to the separation of frontier orbitals, resulting in a small Δ*E*_ST_. In addition, intermolecular C–H···π interactions are also observed (Figure S1), which are conducive to increasing the structural rigidity of the molecules and restraining intramolecular vibration that expedites non-radiative decay.

To gain insight into the molecular geometries and frontier orbital distributions, density functional theory (DFT) calculations were performed on these HPB-based molecules. The HOMO are mainly distributed on the electron-donating 10-phenyl-phenoxazine (PXZ) and DMAC moieties, while the LUMO are located on the electron-accepting TRZ moiety and partially extend to the central phenyl ring of HPB (Figure 2B). On one hand, the phenyls in HPB are aligned tightly in a highly twisted molecular conformation, resulting in the separation of HOMO and LUMO. On the other hand, the molecular orbitals are spatially proximate, allowing through-space charge transfer process to occur, and thus enhance the radiative decay rate. As a result, very small Δ*E*_ST_ and high PLQY can be expected, which make these materials promising candidates for efficient emitters. According to the time-dependent density functional theory (TD-DFT) calculations, the Δ*E*_ST_ values of TRZ-HPB-PXZ and TRZ-HPB-DMAC are estimated to be 0.0017 and 0.0013 eV, respectively, which are small enough for RISC process.

**Figure 3:**
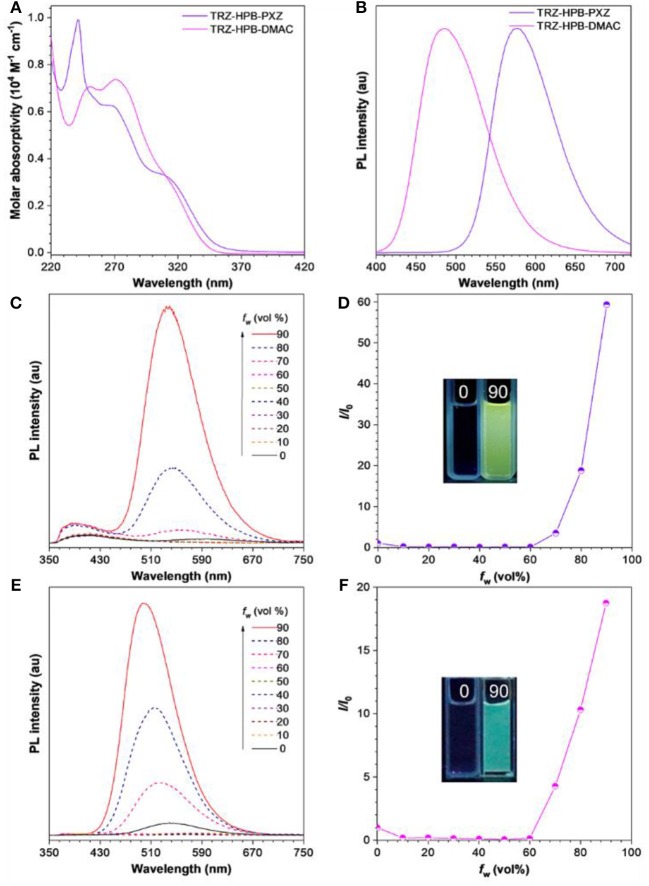
**(A)** Absorption spectra of these HPB-based molecules in THF solutions (10^−5^ M). **(B)** PL spectra of these HPB-based molecules in neat films. PL spectra of **(C)** TRZ-HPB-PXZ, **(E)** TRZ-HPB-DMAC in THF/water mixtures with different water fractions (*f*_w_), Plots of *I*/*I*_0_ vs. *f*_w_ of **(D)** TRZ-HPB-PXZ, **(F)** TRZ-HPB-DMAC. *I*_0_ is the PL intensity in pure THF. Inset: photographs of these HPB-based molecules in THF/water mixtures (*f*_w_ = 0 and 90%), taken under 310 nm excitation.

The absorption and PL spectra of these HPB-based molecules are depicted in Figure 3. There are distinct broad absorption shoulders in the range of 300–350 nm, which are associated with the intramolecular charge transfer (ICT), as confirmed by theoretical calculation (Figure S2). Their PL spectra in dilute THF solutions show two peaks. The weak PL bands at 370–390 nm are mainly attributable to the localized state emissions of donor and acceptor moieties (Kubota et al., 2014), while the strong PL peaks at 595 nm for TRZ-HPB-PXZ and 541 nm for TRZ-HPB-DMAC are assigned to the ICT state emissions of the molecules. Similar dual emissions have also been discovered by other research groups (Lee et al., 2015b; Shiu et al., 2017). The PL spectra display an apparent solvatochromic effect in different solvents (Figure S3). For example, the maximum PL peak of TRZ-HPB-PXZ shifts from 457 nm in hexane to 595 nm in THF. Analogous spectral movements are also found in TRZ-HPB-DMAC, indicative of their strong ICT characters. Both HPB-based molecules exhibit weak emissions in THF solutions with low PLQY of 5.5 and 9.1% (Table 1). However, their emissions are significantly enhanced when fabricated into neat films. The PL peak of TRZ-HPB-PXZ is located at around 576 nm and that of TRZ-HPB-DMAC is blue-shifted to 484 nm. High PLQY of 61.5 and 51.8% are recorded in neat films of TRZ-HPB-PXZ and TRZ-HPB-DMAC, respectively, which are improved by about one order of magnitude compared with those in solutions. The obvious increase in PLQY of TRZ-HPB-PXZ and TRZ-HPB-DMAC in neat films indicates that they should possess AIE character. To further corroborate the AIE nature of TRZ-HPB-PXZ and TRZ-HPB-DMAC, their PL spectra in THF/water mixtures were measured. It can be seen that along with the nanoaggregates formation by adding a large amount of water into THF solutions, the emissions of TRZ-HPB-PXZ and TRZ-HPB-DMAC are enhanced significantly (Figure 3), clearly validating the AIE nature (Mei et al., 2015).

**Table 1:** Photophysical property of TRZ-HPB-PXZ and TRZ-HPB-DMAC.

	**Solution^a^**				**Neat film^b^**							
	**λ_**abs**_ [nm]**	**λ_**em**_ [nm]**	**$\Phi_{\text{F}}^{\text{c}}$ [%]**	** < τ>^d^ [ns]**	**λ_**em**_ [nm]**	**$\Phi_{\text{F}}^{\text{c}}$ [%]**	** < τ>^d^ [ns]**	**$\tau_{\text{prompt}}^{\text{e}}$ [ns]**	**$\tau_{\text{delayed}}^{\text{e}}$ [μs]**	**$R_{\text{delayed}}^{\text{f}}$ [%]**	**$k_{\text{RISC}}^{\text{g}}$ [ × 10^**5**^s^**−1**^]**	**$\Delta E_{\text{ST}}^{\text{h}}$ [eV]**
TRZ-HPB-PXZ	310	595	5.5	15.2	576	61.5	1798.4	41.6	2.1	84.8	31.0	0.02
TRZ-HPB-DMAC	310	541	9.1	357.5	484	51.8	3354.8	48.4	4.7	70.6	7.2	0.09

a *Measured in THF solution (10^−5^ M) at room temperature*.

b *Vacuum-deposited on a quartz substrate. ^c^Determined by a calibrated integrating sphere under nitrogen at room temperature*.

d *Mean fluorescence lifetime evaluated at 300 K under nitrogen. ^e^Fluorescence lifetimes of prompt (τ_prompt_) and delayed (τ_delayed_) components evaluated at 300 K under nitrogen. ^f^Ratio of delayed component. ^g^Rate constant of RISC calculated from the equations given in the Supplementary Material. ^h^Estimated from the high-energy onsets of fluorescence and phosphorescence spectra at 77 K*.

**Figure 4:**
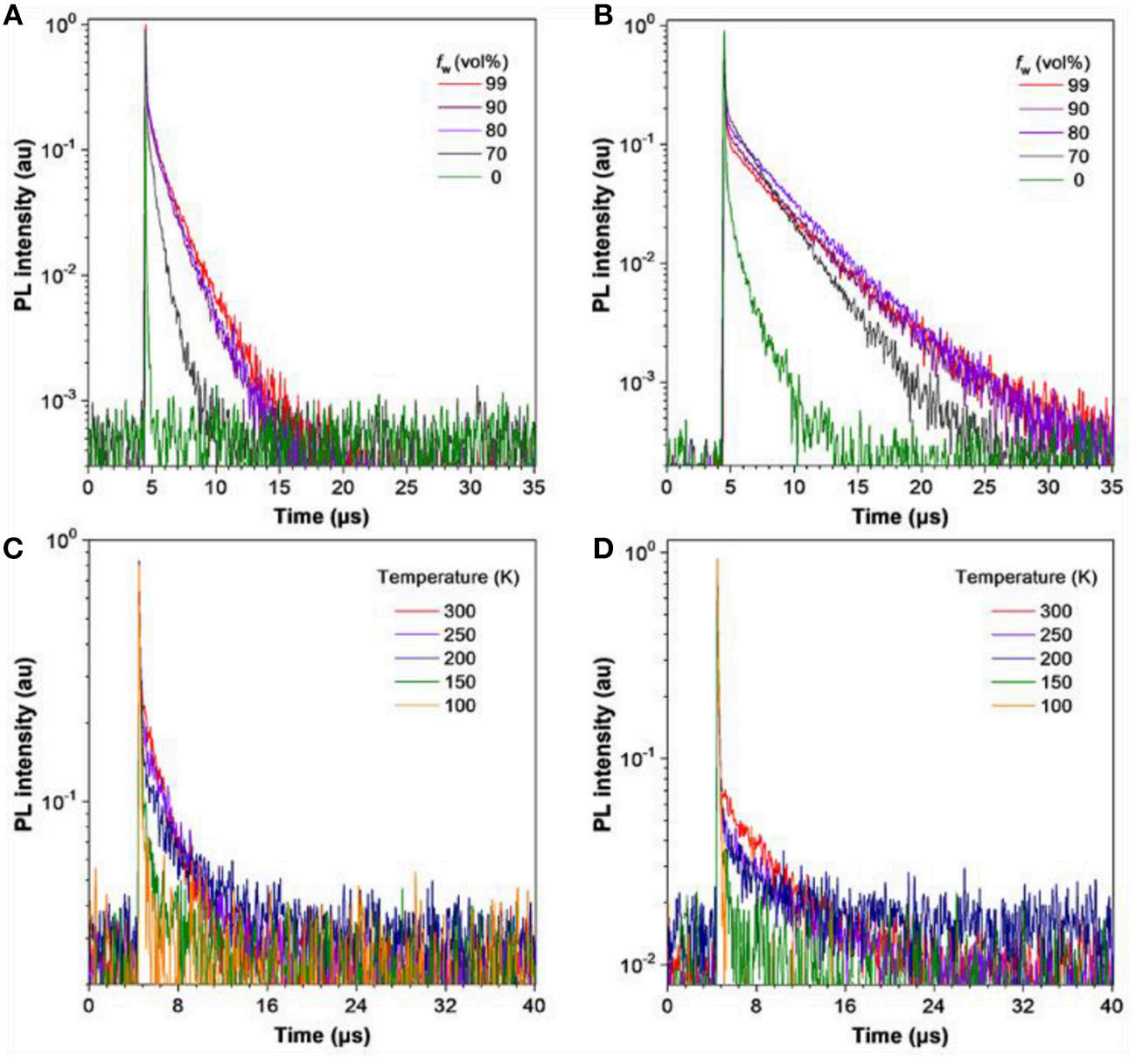
Transient PL decay spectra of **(A)** TRZ-HPB-PXZ and **(B)** TRZ-HPB-DMAC in THF/water mixtures with different water fractions (*f*_w_). Temperature dependent PL transient decay spectra of neat films of **(C)** TRZ-HPB-PXZ, and **(D)** TRZ-HPB-DMAC, measured under nitrogen.

To deepen the understanding of their PL properties, the transient PL decay spectra in THF/water mixtures and neat films were measured (Figure 4). Their PL decay profiles consist of two parts: a nanosecond-scale component and a microsecond-scale one, which can be attributed to prompt fluorescence and delayed fluorescence, respectively. In THF solution, TRZ-HPB-PXZ and TRZ-HPB-DMAC possess short mean lifetimes of 15.2 and 357.5 ns, respectively, and the delayed components are hardly recognizable (Table 1). However, prominent delayed components are discovered upon aggregate formation (Table S1), revealing that the delayed fluorescence is induced by aggregation (Aizawa et al., 2017; Gan et al., 2017; Guo et al., 2017a, 2018a; Huang et al., 2017; Liu et al., 2018). The neat films of TRZ-HPB-PXZ and TRZ-HPB-DMAC show long lifetimes in microsecond scale (1.8 and 3.4 μs), which are significantly longer than those recorded in solutions (Table S2), further demonstrating their AIDF attributes. When dispersed in good solvents, the intramolecular rotational, and vibrational motions are spiritedly active, and as a result, excited state energy is dissipated in a non-radiative manner via internal conversion (IC) (Li and Li, 2017), leading to faint emission. Besides, rapid IC process will impede intersystem crossing (ISC) and RISC processes, which is responsible for the indiscernible delayed fluorescence. Whereas, in aggregates, the molecular motions are restricted greatly because of the limited physical space, resulting in blocking of non-radiative IC channels and promotion of ISC and RISC processes under the basis of small Δ*E*_ST_. Therefore, the molecules can emit strong emission with prominent delayed component (Fan et al., 2017; Guo et al., 2018b). The fluorescence decay is positively correlated with temperature and the long-lived component is promoted as temperature increases (Table S3). The Δ*E*_ST_ values of these molecules were obtained from the singlet and triplet energies calculated from the onset of the fluorescence and phosphorescence spectra, respectively, measured in neat films at 77 K (Figure S4). TRZ-HPB-PXZ and TRZ-HPB-DMAC exhibit small Δ*E*_ST_ values of 0.02 and 0.09 eV coupled with fast RISC rate constants (*k*_RISC_) of 3.1 × 10^6^ and 7.2 × 10^5^ s^−1^, respectively (Table S4), which are favorable for the occurrence of delayed fluorescence.

**Figure 5:**
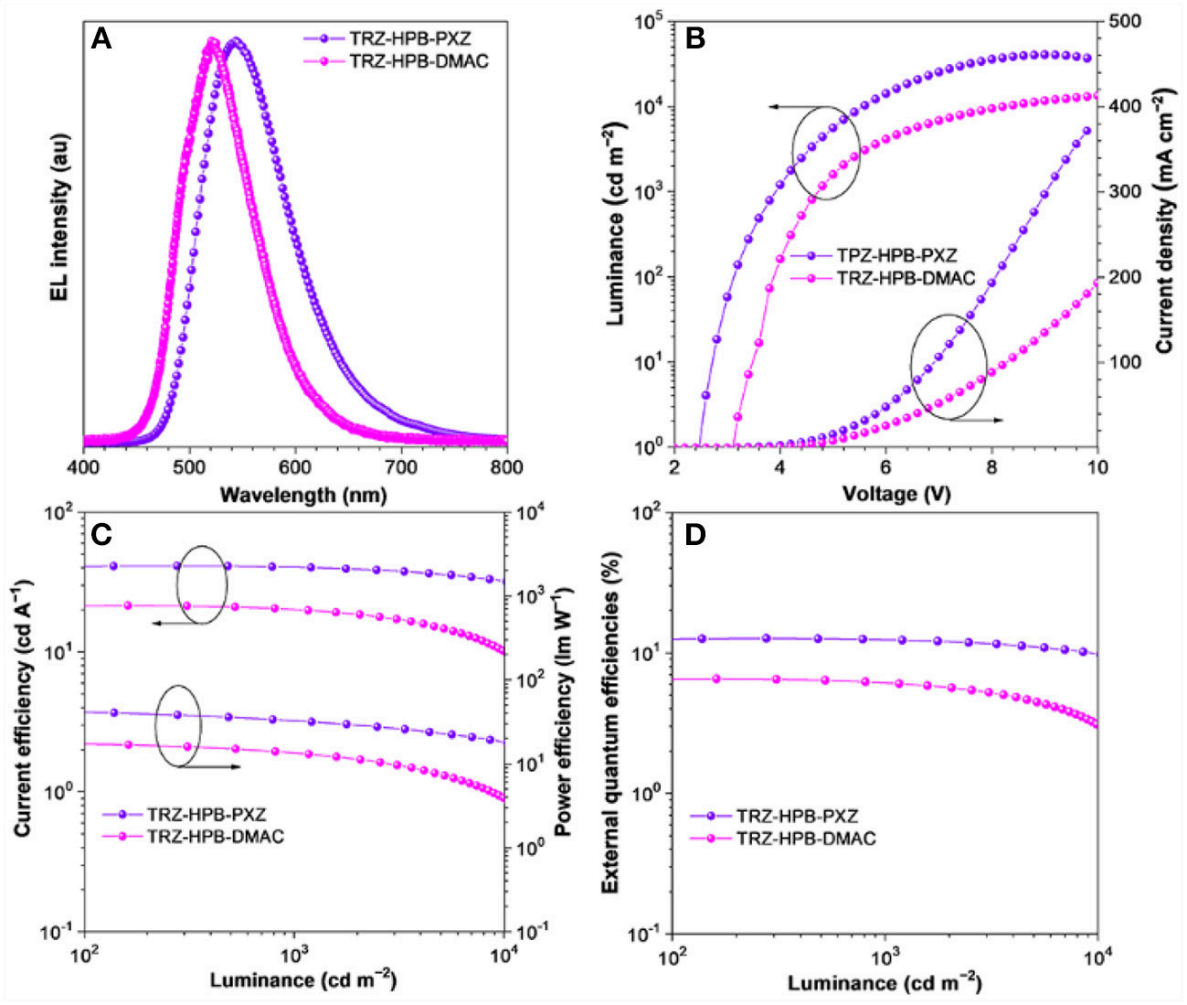
**(A)** EL spectra at luminance of 1,000 cd m^−2^, and characteristic curves of **(B)** luminance-voltage-current density, **(C)** current efficiency-luminance-power efficiency, and **(D)** external quantum efficiency-luminance of the non-doped OLEDs.

In view of excellent solid-state PL efficiencies, prominent delayed fluorescence as well as good thermal stability of TRZ-HPB-PXZ and TRZ-HPB-DMAC, multilayer non-doped OLEDs were fabricated to investigate their EL properties. The configurations of devices were: ITO/HATCN (5 nm)/TAPC (20 nm)/TCTA (5 nm)/emitter/TmPyPB (55 nm)/LiF (1 nm)/Al, in which the neat films of TRZ-HPB-PXZ (35 nm) (device I) and TRZ-HPB-DMAC (20 nm) (device II) acted as emitters; dipyrazino[2,3-f:2′,3′-h]quinoxaline-2,3,6,7,10,11-hexacarbonitrile (HATCN), 1,1′-bis(di-4-tolylaminophenyl)cyclohexane (TAPC), 1,3,5-tri(mpyrid-3-yl-phenyl)benzene (TmPyPB) and 4,4′,4″-tris(carbazol-9-yl)-triphenylamine (TCTA) functioned as hole injection, hole-transporting, electron-transporting and exciton-blocking layers, respectively. Appropriate adjustment of the emitter thickness had been made in order to achieve better device performance. The character curves are presented in Figure 5 and the key data of the devices are listed in Table 2. Devices I and II are turned on at a low voltage of 2.5 and 3.1 V, respectively, implying efficient carrier injection and transport into the emitters. Devices I emits bright yellow light (λ_EL_ = 544 nm) with color coordinates of CIE_x, y_ (0.39, 0.57). Its maxima current (η_C_), power (η_P_) and external quantum (η_ext_) efficiencies are 41.2 cd A^−1^, 44.9 lm W^−1^, and 12.7%, respectively. More significantly, this non-doped device of TRZ-HPB-PXZ enjoys excellent efficiency stability. When the luminance is increased to 1,000 cd m^−2^, the η_C_, η_P_, and η_ext_ still remain as 40.1 cd A^−1^, 31.5 lm W^−1^, and 12.3%, respectively. The roll-off of current efficiency is only 2.7%. The EL peak of device II is located at 521 nm (CIE_x, y_ = 0.28, 0.58) and the maximum η_ext_ is 6.5%. The inferior EL performance may be due to the lower PLQY and increased Δ*E*_ST_ of TRZ-HPB-DMAC compared with those of TRZ-HPB-PXZ. It is noted that the EL spectra are shifted relative to their PL spectra in films. Similar differences are reported by other groups, which may be caused by optical microcavity effect or different excited dipole moments of the molecules under electrical excitation and photoexcitation (Chen et al., 2014; Sun et al., 2015; Zhang et al., 2016).

**Table 2:** EL performances of the non-doped OLEDs based on HPB-based molecules^a^.

**Device**	***V*_**on**_ [V]**	**Maximum values**				**Values at 1,000 cd m**^****−2****^						
		**η_**C**_ [cd A^**−1**^]**	**η_**P**_ [lm W^**−1**^]**	**η _**ext**_ [%]**	***L* [cd m^**−2**^]**	***V* [V]**	**η _**C**_ [cd A^**−1**^]**	**η _**P**_ [lm W^**−1**^]**	**η _**ext**_ [%]**	**RO [%]**	**λ _**EL**_ [nm]**	**CIE (x, y)**
I	2.5	41.2	44.9	12.7	40,382	4.0	40.1	31.5	12.3	2.7	544	(0.39, 0.57)
II	3.1	21.4	17.6	6.5	15,460	4.8	19.9	13.0	6.0	7.0	521	(0.28, 0.58)

a *V_on_, turn-on voltage at 1 cd m^−2^; η_C_, current efficiency; η_P_, power efficiency; η_ext_, external quantum efficiency; L, luminance; CIE, Commission International de I′Eclairage coordinates; λ_EL_, maxima of electroluminescent spectra; RO, current efficiency roll-off from maximum value to that at 1,000 cd m^−2^. Emitter: TRZ-HPB-PXZ (device I); TRZ-HPB-DMAC (device II)*.

Theoretical maximum η_ext_ was also calculated in order to evaluate the exciton utilization of TRZ-HPB-PXZ-based device I. According to the values of entire PLQY and lifetime, the PLQY contributed by prompt fluorescence (Φ_prompt_) and delayed fluorescence (Φ_delayed_) of TRZ-HPB-PXZ neat film are 9.4 and 52.1% (Table S4), respectively. Assuming that the out-coupling efficiency is 20–30% and the charge transport is balanced, the maximum theoretical η_ext_ calculated from photophysical data of TRZ-HPB-PXZ is 12.3–18.5% (Park et al., 2016), which is reasonable according to the experimental data, indicating that the triplet excitons have been sufficiently converted to radiative singlet excitons. However, the experimental η_ext_ of TRZ-HPB-DMAC is inferior to the maximum theoretical η_ext_ (10.3–15.5%). The phenomenon may be attributed to the poorer triplet-to-singlet conversion efficiency of TRZ-HPB-DMAC in EL devices due to the lower *k*_RISC_, which is about one-fifth of TRZ-HPB-PXZ′s *k*_RISC_. In addition, the different carrier transport capabilities of PXZ and DMAC moieties will also influence the carrier balance in EL devices, and thus affect devices′ performance. These results demonstrate that through-space charge transfer contributes to a small Δ*E*_ST_ and thus delayed fluorescence, which allows a high exciton utilization of the emitters. In addition, the intriguing AIDF properties are conductive to suppressing emission quenching and exciton annihilation in neat films. The synergistic effect of these factors ensures non-doped OLEDs with high efficiency and small efficiency roll-off.

## Conclusions

In summary, two HPB-based luminogens TRZ-HPB-PXZ and TRZ-HPB-DMAC with good thermal and electrochemical stabilities are synthesized and characterized. Both luminogens exhibit through-space charge transfer feature between D and A moieties, which brings about efficient separation of HOMO and LUMO, and thus small Δ*E*_ST_. TRZ-HPB-PXZ and TRZ-HPB-DMAC are barely fluorescent in solutions but show strong emissions with prominent delayed fluorescence in the aggregated state, that is AIDF property. Their neat films have distinct delayed fluorescence and high PLQY. Non-doped OLEDs using these luminogens as light-emitting layers have achieved excellent performance with a maximum η_ext_ of 12.7% and very small efficiency roll-off of 2.7% at 1,000 cd m^−2^. These results reveal that AIDF luminogens with through-space charge transfer can realize high exciton utilization and suppressed exciton annihilation at high luminance, which could be promising candidates for OLEDs with improved efficiency and stability.

## Author Contributions

All authors listed have made a substantial, direct and intellectual contribution to the work, and approved it for publication.

### Conflict of Interest Statement

The authors declare that the research was conducted in the absence of any commercial or financial relationships that could be construed as a potential conflict of interest.

## References

[B1] Adachi C. Baldo M. A. Thompson M. E. Forrest S. R. (2001). Nearly 100% internal phosphorescence efficiency in an organic light-emitting device. J. Appl. Phys. 90:5048 10.1063/1.1409582

[B2] Aizawa N. Tsou C.-J. Park I. S. Yasuda T. (2017). Aggregation-induced delayed fluorescence from phenothiazine-containing donor-acceptor molecules for high-efficiency non-doped organic light-emitting diodes. Polym. J. 49, 197–202. 10.1038/pj.2016.82

[B3] Baldo M. A. O′Brien D. F. You Y. Shoustikov A. Sibley S. Thompson M. E. (1998). Highly efficient phosphorescent emission from organic electroluminescent devices. Nature 395, 151–154. 10.1038/25954

[B4] Balzani V. Clemente-León M. Credi A. Lowe J. N. Badjić J. D. Stoddart J. F.. (2003). Controlling multivalent interactions in triply-threaded two-component superbundles. Chem. Eur. J. 9, 5348–5360. 10.1002/chem.200304979 14613145

[B5] Cao X. Zhang D. Zhang S. Tao Y. Huang W. (2017). CN-Containing donor-acceptor-type small-molecule materials for thermally activated delayed fluorescence OLEDs. J. Mater. Chem. C 5, 7699–7714. 10.1039/c7tc02481a

[B6] Chen L. Jiang Y. Nie H. Hu R. Kwok H. S. Huang F.. (2014). Rational design of aggregation-induced emission luminogen with weak electron donor–acceptor interaction to achieve highly efficient undoped bilayer OLEDs. ACS Appl. Mater. Interfaces 6, 17215–17225. 10.1021/am505036a 25254940

[B7] Chen X. L. Jia J. H. Yu R. Liao J. Z. Yang M. X. Lu C. Z. (2017). Combining charge-transfer pathways to achieve unique thermally activated delayed fluorescence emitters for high-performance solution-processed, non-doped blue OLEDs. Angew. Chem. Int. Ed. 56, 15006–15009. 10.1002/anie.201709125 28990260

[B8] Cho S. Li W. S. Yoon M. C. Ahn T. K. Jiang D. L. Kim J.. (2006). Relationship between incoherent excitation energy migration processes and molecular structures in Zinc (II) porphyrin dendrimers. Chem. Eur. J. 12, 7576–7584. 10.1002/chem.200600213 16927274

[B9] Endo A. Ogasawara M. Takahashi A. Yokoyama D. Kato. Y Adachi C. (2009). Thermally activated delayed fluorescence from Sn^4+^-porphyrin complexes and their application to organic light emitting diodes–A novel mechanism for electroluminescence. Adv. Mater. Weinheim. 21, 4802–4806. 10.1002/adma.200900983 21049498

[B10] Fan J. Lin L. Wang C.-K. (2017). Excited state properties of non-doped thermally activated delayed fluorescence emitters with aggregation-induced emission: a QM/MM study. J. Mater. Chem. C 5, 8390–8399. 10.1039/c7tc02541f

[B11] Furue R. Nishimoto T. Park I. S. Lee J. Yasuda T. (2016). Aggregation-induced delayed fluorescence based on donor/acceptor-tethered janus carborane triads: unique photophysical properties of non-doped OLEDs. Angew. Chem. Int. Ed. 55, 7171–7175. 10.1002/anie.201603232 27145481

[B12] Gan S. Luo W. He B. Chen L. Nie H. Hu R. (2016). Integration of aggregation-induced emission and delayed fluorescence into electronic donor-acceptor conjugates. J. Mater. Chem. C 4, 3705–3708. 10.1039/c5tc03588k

[B13] Gan S. Zhou J. Smith T. A. Su H. Luo W. Hong Y. (2017). New AIEgens with delayed fluorescence for fluorescence imaging and fluorescence lifetime imaging of living cells. Mater. Chem. Front. 1, 2554–2558. 10.1039/c7qm00286f

[B14] Geng Y. Fechtenkötter A. Müllen K. (2001). Star-like substituted hexaarylbenzenes: synthesis and mesomorphic properties. J. Mater. Chem. 11, 1634–1641. 10.1039/b101163o

[B15] Gong S. Chen Y. Luo J. Yang C. Zhong C. Qin J. (2011). Bipolar tetraarylsilanes as universal hosts for blue, green, orange, and white electrophosphorescence with high efficiency and low efficiency roll-off. Adv. Funct. Mater. 21, 1168–1178. 10.1002/adfm.201002066

[B16] Guo J. Fan J. Lin L. Zeng J. Liu H. Wang C. K.. (2018b). Mechanical insights into aggregation-induced delayed fluorescence materials with anti-kasha behavior. Adv. Sci. 6:1801629. 10.1002/advs.201801629 30775236 PMC6364497

[B17] Guo J. Li X.-L. Nie H. Luo W. Gan S. Hu S. (2017a). Achieving high-performance non-doped OLEDs with extremely small efficiency roll-off by combining aggregation-induced emission and thermally activated delayed fluorescence. Adv. Funct. Mater. 27:1606458 10.1002/adfm.201606458

[B18] Guo J. Li X.-L. Nie H. Luo W. Hu R. Qin A. (2017b). Robust luminescent materials with prominent aggregation-induced emission and thermally activated delayed fluorescence for high-performance organic light-emitting diodes. Chem. Mater. 29, 3623–3631. 10.1021/acs.chemmater.7b00450

[B19] Guo J. Zhao Z. Tang B. Z. (2018a). Purely organic materials with aggregation-induced delayed fluorescence for efficient nondoped OLEDs. Adv. Opt. Mater. 6:1800264 10.1002/adom.201800264

[B20] Hirata S. Sakai Y. Masui K. Tanaka H. Lee S. Y. Nomura H.. (2015). Highly efficient blue electroluminescence based on thermally activated delayed fluorescence. Nat. Mater. 14, 330–336. 10.1038/nmat4154 25485987

[B21] Huang J. Nie H. Zeng J. Zhuang Z. Gan S. Cai Y. (2017). Highly efficient non-doped OLEDs with negligible efficiency roll-off fabricated from aggregation-induced delayed fluorescence luminogens. Angew. Chem. Int. Ed. 56, 12971–12976. 10.1002/anie.201706752 28833917

[B22] Kaji H. Suzuki H. Fukushima T. Shizu K. Suzuki K. Kubo S.. (2015). Purely organic electroluminescent material realizing 100% conversion from electricity to light. Nat. Commun. 6:8476. 10.1038/ncomms9476 26477390 PMC4634127

[B23] Kanibolotsky A. L. Perepichka I. F. Skabara P. J. (2010). Star-shaped π-conjugated oligomers and their applications in organic electronics and photonics. Chem. Soc. Rev. 39, 2695–2728. 10.1039/b918154g 20520881

[B24] Kawasumi K. Wu T. Zhu T. Chae H. S. Van Voorhis T. Baldo M. A.. (2015). Thermally activated delayed fluorescence materials based on homoconjugation effect of donor-acceptor triptycenes. J. Am. Chem. Soc. 137, 11908–11911. 10.1021/jacs.5b07932 26367852

[B25] Kubota Y. Sakuma Y. Funabiki K. Matsui M. (2014). Solvatochromic fluorescence properties of pyrazine-boron complex bearing a β-Iminoenolate ligand. J. Phys. Chem. A 118, 8717–8729. 10.1021/jp506680g 25171168

[B26] Lambert C. (2005). Hexaarylbenzenes–prospects for toroidal delocalization of charge and energy. Angew. Chem. Int. Ed. 44, 7337–7339. 10.1002/anie.200502105 16206312

[B27] Lambert C. Ehbets J. Rausch D. Steeger M. (2012). Charge-transfer interactions in a multichromophoric hexaarylbenzene containing pyrene and triarylamines. J. Org. Chem. 77, 6147–6154. 10.1021/jo300924x 22731634

[B28] Lee D. R. Kim B. S. Lee C. W. Im Y. Yook K. S. Hwang S.-H.. (2015a). Above 30% external quantum efficiency in green delayed fluorescent organic light-emitting diodes. ACS Appl. Mater. Interfaces 7, 9625–9629. 10.1021/acsami.5b01220 25924007

[B29] Lee J. Shizu K. Tanaka H. Nakanotani H. Yasuda T. Kaji H. (2015b). Controlled emission colors and singlet-triplet energy gaps of dihydrophenazine-based thermally activated delayed fluorescence emitters. J. Mater. Chem. C 3, 2175–2181. 10.1039/c4tc02530j

[B30] Li Q. Li Z. (2017). The strong light-emission materials in the aggregated state: what happens from a single molecule to the collective group. Adv. Sci. 4:1600484. 10.1002/advs.201600484 28725526 PMC5515118

[B31] Liu H. Zeng J. Guo J. Nie H. Zhao Z. Tang B. Z. (2018). High-performance non-doped OLEDs with nearly 100 % exciton use and negligible efficiency roll-off. Angew. Chem. Int. Ed. 130, 9434–9438. 10.1002/ange.201802060 29856500

[B32] Mei J. Leung N. L. C. Kwok R. T. K. Lam J. W. Y. Tang B. Z. (2015). Aggregation-induced emission: together we shine, united we soar! Chem. Rev. 115, 11718–11940. 10.1021/acs.chemrev.5b00263 26492387

[B33] Minaev B. Baryshnikov G. Agren H. (2014). Principles of phosphorescent organic light emitting devices. Phys. Chem. Chem. Phys. 16, 1719–1758. 10.1039/c3cp53806k 24346346

[B34] Park I. S. Lee S. Y. Adachi C. Yasuda T. (2016). Full-color delayed fluorescence materials based on wedge-shaped phthalonitriles and dicyanopyrazines: systematic design, tunable photophysical properties, and OLED performance. Adv. Funct. Mater. 26, 1813–1821. 10.1002/adfm.201505106

[B35] Rajamalli P. Senthilkumar N. Gandeepan P. Huang P. Y. Huang M. J. Ren-Wu C. Z.. (2016). A new molecular design based on thermally activated delayed fluorescence for highly efficient organic light emitting diodes. J. Am. Chem. Soc. 138, 628–634. 10.1021/jacs.5b10950 26709617

[B36] Sasabe H. Kido J. (2013). Recent progress in phosphorescent organic light-emitting devices. Eur. J. Org. Chem. 2013 7653–7663. 10.1002/ejoc.201300544

[B37] Seino Y. Inomata S. Sasabe H. Pu Y. J. Kido J. (2016). High-performance green OLEDs using thermally activated delayed fluorescence with a power efficiency of over 100 lm W^−1^. Adv. Mater. Weinheim. 28, 2638–2643. 10.1002/adma.201503782 26833580

[B38] Shao S. Hu J. Wang X. Wang L. Jing X. Wang F. (2017). Blue thermally activated delayed fluorescence polymers with non-conjugated backbone and through-space charge transfer effect. J. Am. Chem. Soc. 139, 17739–17742. 10.1021/jacs.7b10257 29149569

[B39] Shiu Y.-J. Chen Y.-T. Lee W.-K. Wu C.-C. Lin T.-C. Liu S.-H. (2017). Efficient thermally activated delayed fluorescence of functional phenylpyridinato boron complexes and high performance organic light-emitting diodes. J. Mater. Chem. C 5, 1452–1462. 10.1039/c6tc04994j

[B40] Sun D. Rosokha S. V. Kochi J. K. (2005). Through-space (cofacial) π-delocalization among multiple aromatic centers: toroidal conjugation in hexaphenylbenzene-like radical cations. Angew. Chem. Int. Ed. 44, 5133–5136. 10.1002/anie.200501005 16010702

[B41] Sun J. W. Baek J. Y. Kim K.-H. Moon C.-K. Lee J.-H. Kwon S.-K. (2015). Thermally activated delayed fluorescence from azasiline based intramolecular charge-transfer emitter (DTPDDA) and a highly efficient blue light emitting diode. Chem. Mater. 27, 6675–6681. 10.1021/acs.chemmater.5b02515

[B42] Sun J. W. Lee J.-H. Moon C.-K. Kim K.-H. Shin H. Kim J.-J. (2014). A fluorescent organic light-emitting diode with 30% external quantum efficiency. Adv. Mater. Weinheim. 26, 5684–5688. 10.1002/adma.201401407 24890507

[B43] Tanaka Y. Koike T. Akita M. (2010). 2-Dimensional molecular wiring based on toroidal delocalization of hexaarylbenzene. Chem. Commun. 46, 4529–4531. 10.1039/c0cc00128g 20485728

[B44] Tang C. W. VanSlyke S. A. (1987). Organic electroluminescent diodes. Appl. Phys. Lett. 51:913 10.1063/1.98799

[B45] Tao Y. Yuan K. Chen T. Xu P. Li H. Chen R.. (2014). Thermally activated delayed fluorescence materials towards the breakthrough of organoelectronics. Adv. Mater. Weinheim. 26, 7931–7958. 10.1002/adma.201402532 25230116

[B46] Tsujimoto H. Ha D. G. Markopoulos G. Chae H. S. Baldo M. A. Swager T. M. (2017). Thermally activated delayed fluorescence and aggregation induced emission with through-space charge transfer. J. Am. Chem. Soc. 139, 4894–4900. 10.1021/jacs.7b00873 28345346

[B47] Vij V. Bhalla V. Kumar M. (2016). Hexaarylbenzene: evolution of properties and applications of multitalented scaffold. Chem. Rev. 116, 9565–9627. 10.1021/acs.chemrev.6b00144 27498592

[B48] Waldvogel S. R. Wartini A. R. Rasmussen P. H. Jr. (1999). A triphenylene scaffold with C_3v_-symmetry and nanoscale dimensions. Tetrahedron Lett. 40, 3515–3518. 10.1016/s0040-4039(99)00545-6

[B49] Wang H. Xie L. Peng Q. Meng L. Wang Y. Yi Y.. (2014). Novel thermally activated delayed fluorescence materials-thioxanthone derivatives and their applications for highly efficient OLEDs. Adv. Mater. Weinheim. 26, 5198–5204. 10.1002/adma.201401393 24903266

[B50] Zhang J. Ding D. Wei Y. Xu H. (2016). Extremely condensing triplet states of DPEPO-type hosts through constitutional isomerization for high-efficiency deep-blue thermally activated delayed fluorescence diodes. Chem. Sci. 7, 2870–2882. 10.1039/c5sc04848f. 30090280 PMC6054027

[B51] Zhang Q. Li B. Huang S. Nomura H. Tanaka H. Adachi C. (2014). Efficient blue organic light-emitting diodes employing thermally activated delayed fluorescence. Nat. Photonics 8, 326–332. 10.1038/nphoton.2014.12

[B52] Zhen S. Mao J. C. Chen L. Ding S. Luo W. Zhou X. S.. (2018). Remarkable multichannel conductance of novel single-molecule wires built on through-space conjugated hexaphenylbenzene. Nano Lett. 18, 4200–4205. 10.1021/acs.nanolett.8b01082 29911870

