# Supplementary material for: New Aggregation-Induced Delayed Fluorescence Luminogens With Through-Space Charge Transfer for Efficient Non-doped OLEDs
Source: Front Chem. 2019 Apr 5;7:199. doi: 10.3389/fchem.2019.00199 (PMC6460965; doi:10.3389/fchem.2019.00199)
Supplement: Supplementary file 1 [file Data_Sheet_1.pdf]

Supplementary Material

New Aggregation-Induced Delayed Fluorescence Luminogens with Through-Space Charge Transfer for Efficient Nondoped OLEDs

Panpan Zhang[1], Jiajie Zeng[1], Jingjing Guo[1], Shijie Zhen[1], Biao Xiao[2], Zhiming Wang[1], Zujin Zhao*[1], Ben Zhong Tang*[1,3]

[1] State Key Laboratory of Luminescent Materials and Devices, Center for Aggregation-Induced Emission, South China University of Technology, Guangzhou 510640, China.

[2] Key Laboratory of Optoelectronic Chemical Materials and Devices, Ministry of Education, School of Chemical and Environmental Engineering, Jianghan University, Wuhan 430056, China.

[3] Department of Chemistry, Hong Kong Branch of Chinese National Engineering Research Center for Tissue Restoration and Reconstruction, The Hong Kong University of Science and Technology, Clear Water Bay, Kowloon, Hong Kong, China.

GENERAL

All the chemicals and reagents were purchased from commercial sources and used as received without further purification. The final products were subjected to vacuum sublimation to further improve purity before photoluminescence (PL) and electroluminescence (EL) properties investigations. ^{1}H and ^{13}C NMR spectra were measured on a Bruker AV 500 spectrometer in $CDCl_3$ at room temperature. High resolution mass spectra (HRMS) were recorded on a GCT premier CAB048 mass spectrometer operating in MALDI-TOF mode. Single crystals of TRZ-HPB-DMAC were grown in tetrahydrofuran-hexane mixtures and single crystal X-ray diffraction intensity data were collected at 173 K on a Bruker-Nonices Smart Apex CCD diffractometer with graphite monochromated MoKα radiation. Processing of the intensity data was carried out using the SAINT and SADABS routines, and the structure and refinement were conducted using the SHELTL suite of X-ray programs (version 6.10). UV-vis absorption spectra were measured on a Shimadzu UV-2600 spectrophotometer. PL spectra were recorded on a Horiba Fluoromax-4 spectrofluorometer. PLQY data were measured using a Hamamatsu absolute PL quantum yield spectrometer C11347 Quantaurus_QY. Cyclic voltammetry was measured in a solution of tetra-*n*-butylammonium hexafluorophosphate (Bu_4NPF_6, 0.1 M) in acetonitrile containing at a scan rate of 100 mV s^{-1}. Three-electrode system (Ag/Ag^+, platinum wire and glassy carbon electrode were used as reference, counter and work electrode, respectively) was used in the CV method. HOMO = $-[E_{ox} + 4.8]$ eV, and LUMO = $-[E_{re} + 4.8]$ eV. E_{ox} and E_{re} represent the onset oxidation and reduction potentials relative to Fc/Fc^+, respectively. The ground-state geometries were optimized using the density function theory (DFT) method with BMK functional at the basis set level of 6-31G*, and the ΔE_{ST} values were calculated by time-dependent DFT (TDDFT) method at the BMK/6-31G* level (Fan et al., 2017). All the calculations were performed using Gaussian09 package. Differential scanning calorimetry (DSC) analysis was carried out on a DSC Q1000 under dry nitrogen at a heating rate of 10 ^{o}C min^{-1}, and thermogravimetric analysis was performed on a Netzsch TG 209 F3 under nitrogen with a heating rate of 20 ^{o}C min^{-1}.

ADDITIONAL SPECTRA AND DATA

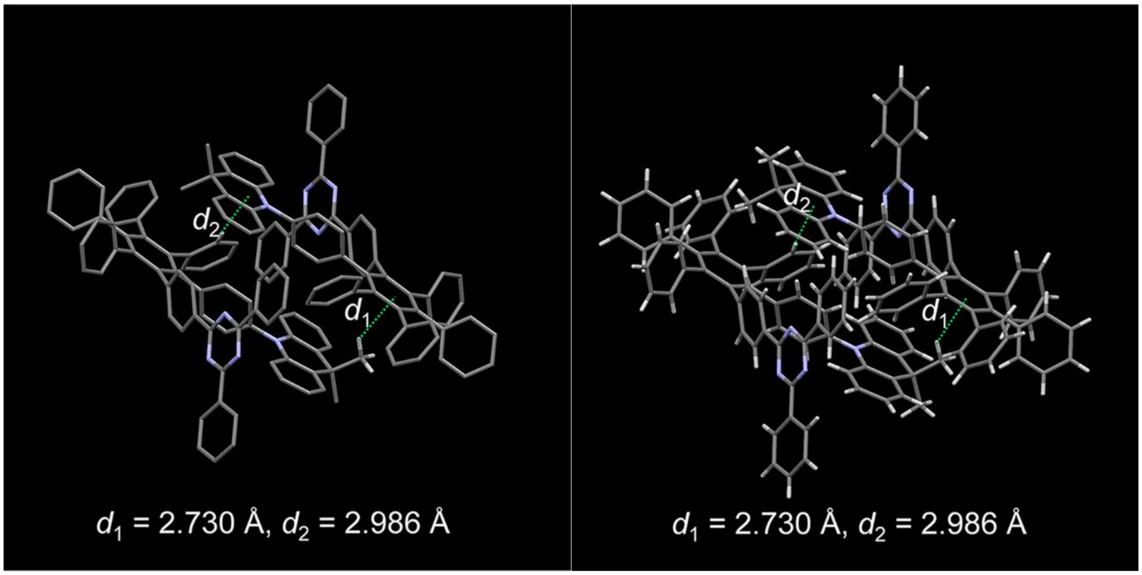

Figure S1. Molecular packing of TRZ-HPB-DMAC in crystals.

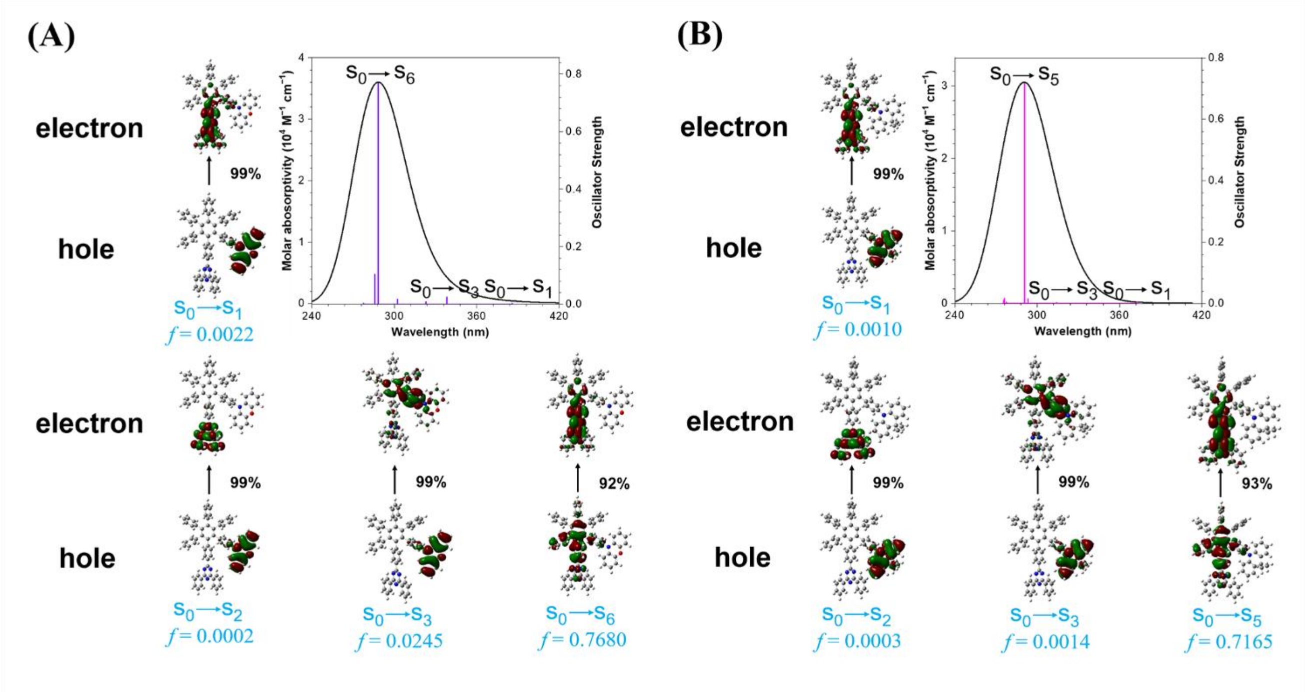

Figure S2. Natural transition orbitals (NTOs) and calculated absorption spectra of of (A) TRZ-HPB-PXZ and (B) TRZ-HPB-DMAC corresponding to the $S_0 \rightarrow S_n$ (ground-state geometry) transitions. The contributeons of the electron and hole excitations to the transitions are indicated. *f* is oscillator strength.

There are distinct broad absorption shoulders in the range of 300–350 nm, in ordre to illuminate the origins of the absorption peaks, natural transition orbitals (NTOs) were calculated and the absorption spectra were analyzed (Lu et al., 2012). As illustrated in **Figure S2A**, the high-energy band (288 nm) in TRZ-HPB-PXZ is attributed to the $S_0 \rightarrow S_6$ transition, which is associated with the locally excited (LE) state, whereas the lower energy transitions including $S_0 \rightarrow S_1$, $S_0 \rightarrow S_2$ and $S_0 \rightarrow S_3$ transitions are obviously assigned to the intramolecular charge transfer (ICT) transition. In other words, the low-

energy band absorption is attributed to ICT transition, corresponding to the broad absorption shoulder in the range of 300–350 nm measured in experiment. Similarly, TRZ-HPB-DMAC shows a high-energy band (291 nm) associated with the $S_0 \rightarrow S_5$ transition related to the LE state, the low-energy band absorption exhibit ICT and agree well with the experimental data (**Figure S2B**). Notably, the oscillator strength of the lower energy transition of TRZ-HPB-PXZ is distinctly stronger than that of TRZ-HPB-DMAC, which is consistent with stronger charge transfer character of TRZ-HPB-PXZ. In addition, we have also found that many other groups attribute the low-energy band absorption to the ICT transition (Chen et al., 2017; Park et al., 2017).

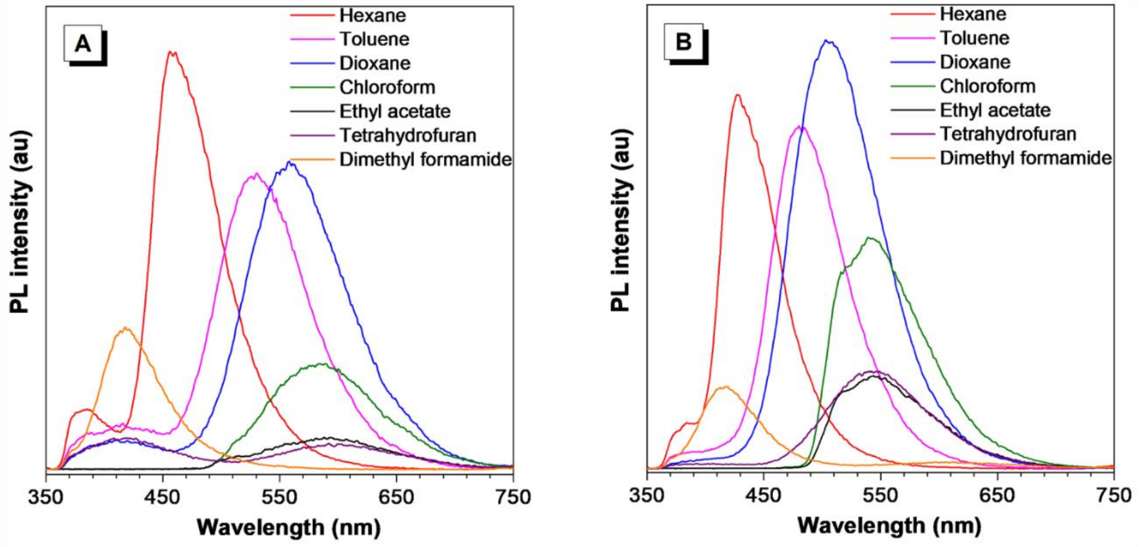

Figure S3. PL spectra of **(A)** TRZ-HPB-PXZ and **(B)** TRZ-HPB-DMAC in various solutions at room temperature.

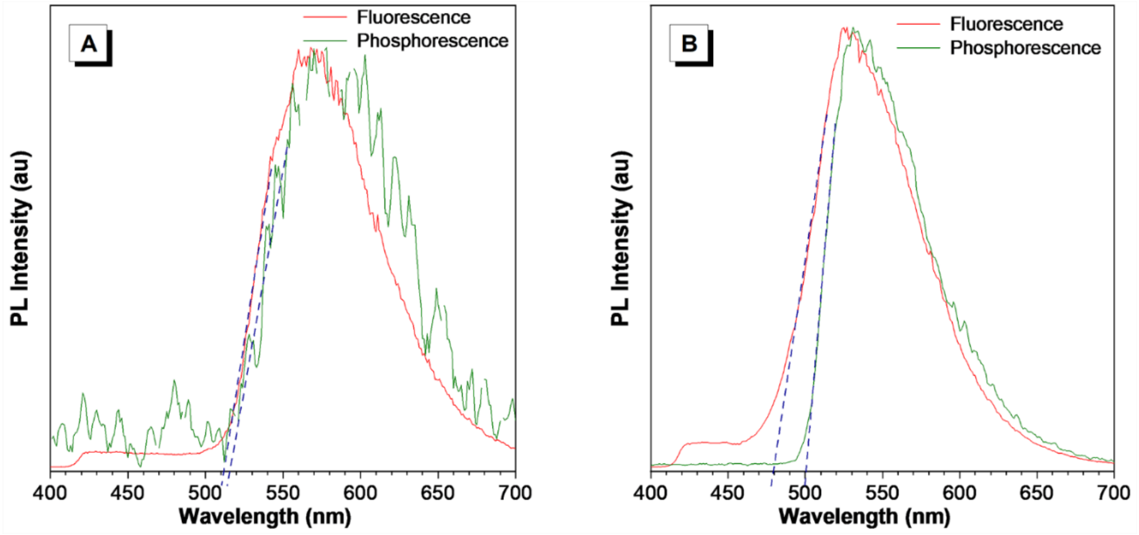

Figure S4. Fluorescence and phosphorescence spectra of neat films of **(A)** TRZ-HPB-PXZ and **(B)** TRZ-HPB-DMAC, measured at 77 K under nitrogen.

Table S1. Transient PL decay data of TRZ-HPB-PXZ and TRZ-HPB-DMAC in THF/water mixtures with different water fractions (f_w).

compound	f_w	$<\tau>$ [ns]	$<\tau_1>$ [ns]	$<\tau_2>$ [ns]	A_1	A_2	R_{prompt} [%]	$R_{delayed}$ [%]
TRZ-HPB-PXZ	0	15.2	14.0	102.6	158956.0	318.9	99	1
	70	382.5	48.1	664.5	9562.1	821.1	46	54
	80	1033.1	56.0	1366.3	18145.9	2180.2	25	75
	90	1009.0	55.3	1368.3	17945.9	1925.3	27	73
	99	1212.3	59.7	1623.3	17756.8	1831.0	26	74
TRZ-HPB-DMAC	0	357.5	53.0	1102.4	20732.6	407.7	71	29
	70	2115.0	66.5	2573.8	16474.5	1899.6	18	82
	80	3094.2	64.3	3715.3	17652.0	1490.9	17	83
	90	2874.8	61.5	3599.6	18740.7	1242.5	20	80
	99	3000.8	59.9	3863.2	19609.3	1037.7	23	77

Table S2. Transient PL decay data of TRZ-HPB-PXZ and TRZ-HPB-DMAC in THF solutions and neat films, measured at 300 K under nitrogen.[a)]

compound	state	$<\tau>$ [ns]	$<\tau_1>$ [ns]	$<\tau_2>$ [ns]	A_1	A_2	R_{prompt} [%]	$R_{delayed}$ [%]
TRZ-HPB-PXZ	THF solution	15.2	14.0	102.6	158956.0	318.9	99	1
	neat film	1798.4	41.6	2114.3	2236.2	244.5	15	85
TRZ-HPB-DMAC	THF solution	357.5	53.0	1102.4	20732.6	407.7	71	29
	neat film	3354.8	48.4	4733.5	7304.2	178.9	29	71

[a)]Transient PL decay data are fitted by multiple-exponential function and the mean fluorescence lifetimes ($<\tau>$) were calculated by $<\tau> = \Sigma A_i\tau_i^2/\Sigma A_i\tau_i$, where A_i is the pre-exponential for lifetime τ_i. R_{prompt} and $R_{delayed}$ are individual component ratio for prompt and delayed fluorescence. $R_{prompt} = \tau_1A_1/(\tau_1A_1 + \tau_2A_2 + \tau_3A_3)$ (Nakagawa et al., 2012), $R_{delayed} = 1 – R_{prompt}$.

Table S3. Transient PL decay data of TRZ-HPB-PXZ and TRZ-HPB-DMAC in neat film at different temperatures.

compound	T [K]	<τ> [ns]	<τ_1> [ns]	<τ_2> [ns]	A_1	A_2	R_{prompt} [%]	$R_{delayed}$ [%]
TRZ-HPB-PXZ	100	159.5	26.9	231.7	1157.0	247.1	35	65
	150	1209.9	106.3	6019.4	11634.4	47.1	81	19
	200	2663.7	100.6	3612.1	1267.2	95.4	27	73
	250	2476.5	59.2	2962.3	1727.8	171.9	17	83
	300	1798.4	41.6	2114.3	2236.2	244.5	15	85
TRZ-HPB-DMAC	100	113.2	21.3	159.0	1254.6	337.5	33	67
	150	784.0	81.9	2133.9	1530.8	30.6	66	34
	200	2284.1	79.7	4552.2	1627.4	27.7	51	49
	250	4387.2	60.9	6480.6	5931.4	115.1	33	67
	300	3354.8	48.4	4733.5	7304.2	178.9	29	71

Table S4. Photophysical data of TRZ-HPB-PXZ and TRZ-HPB-DMAC in neat films. [a)]

	TRZ-HPB-PXZ	TRZ-HPB-DMAC
Φ_{PL} [%]	61.5	51.8
τ_{prompt} [ns]	41.6	48.4
$\tau_{delayed}$ [μs]	2.1	4.7
$R_{delayed}$ [%]	84.8	70.6
Φ_{prompt} [%]	9.4	15.2
$\Phi_{delayed}$ [%]	52.1	36.6
Φ_{ISC} [%]	84.8	70.6
Φ_{RISC} [%]	61.5	51.8
K_F [$\times10^6$ s^{-1}]	2.3	3.2
K_{IC} [$\times10^6$ s^{-1}]	1.4	2.9
K_{ISC} [$\times10^6$ s^{-1}]	20.4	14.6
K_{RISC} [$\times10^5$ s^{-1}]	31.0	7.2

[a] Abbreviations: Φ_{PL} = absolute photoluminescence quantum yield; τ_{prompt} and $\tau_{delayed}$ = lifetimes calculated from the prompt and delayed fluorescence decay, respectively; $R_{delayed}$ = the ratio of delayed components; Φ_{prompt} and $\Phi_{delayed}$ = prompt and delayed components, respectively, determined from the total Φ_{PL} and the proportion of the integrated area of each component in the transient spectra to the total integrated area; Φ_{ISC} = the intersystem crossing quantum yield; k_F = fluorescence decay rate; k_{IC} = internal conversion decay rate from S_1 to S_0; k_{ISC} = intersystem crossing decay rate from S_1 to T_1; k_{RISC} = the rate constant of reverse intersystem crossing process.

$$\Phi_{prompt} = \Phi_{PL}R_{prompt} \quad (1)$$

$$\Phi_{delayed} = \Phi_{PL}R_{delayed} \quad (2)$$

$$k_F = \Phi_{prompt}/\tau_{prompt} \quad (3)$$

$$\Phi_{PL} = k_F/(k_F + k_{IC}) \quad (4)$$

$$\Phi_{prompt} = k_F/(k_F + k_{IC} + k_{ISC}) \quad (5)$$

$$\Phi_{IC} = k_{IC}/(k_F + k_{IC} + k_{ISC}) \quad (6)$$

$$\Phi_{ISC} = k_{ISC}/(k_F + k_{IC} + k_{ISC}) = 1 - \Phi_{prompt} - \Phi_{IC} \quad (7)$$

$$\Phi_{RISC} = \Phi_{delayed}/\Phi_{ISC} \quad (8)$$

$$k_{RISC} = (k_p k_d \Phi_{delayed})/(k_{ISC}\Phi_{prompt}) \quad (9)$$

$$k_p = 1/\tau_{prompt};\ k_d = 1/\tau_{delayed} \quad (10)$$

REFERENCES

Chen, X.-L., Jia, J.-H., Yu, R., Liao, J.-Z., Yang, M.-X., Lu, C.-Z. (2017). Combining charge-transfer pathways to achieve unique thermally activated delayed fluorescence emitters for high-performance solution-processed, non-doped blue OLEDs. *Angew. Chem. Int. Ed.* 56, 15006–15009. doi: 10.1002/anie.201709125

Fan, J., Lin, L., Wang, C.-K. (2017). Excited state properties of non-doped thermally activated delayed fluorescence emitters with aggregation-induced emission: A QM/MM study*. J. Mater. Chem. C* 5, 8390–8399. doi: 10.1039/c7tc02541f

Lu, T., Chen, F. (2012). Multiwfn: A multifunctional wavefunction analyzer. *J. COMPUT. CHEM*. 33, 580–592. doi: 10.1002/jcc.22885

Nakagawa, T., Ku, S.-Y., Wong, K.-T., Adachi, C. (2012). Electroluminescence based on thermally activated delayed fluorescence generated by a spirobifluorene donor-acceptor structure. *Chem. Commun.* 48, 9580–9582. doi: 10.1039/c2cc31468a

Park, H.-J., Han, S. H., Lee. J. Y. (2017). A directly coupled dual emitting core based molecular design of thermally activated delayed fluorescent emitters**.** *J. Mater. Chem. C* 5, 12143–12150. doi: 10.1039/c7tc03133e
